# Should Dogs and Cats be Given as Gifts?

**DOI:** 10.3390/ani3040995

**Published:** 2013-10-16

**Authors:** Emily Weiss, Emily D. Dolan, Laurie Garrison, Julie Hong, Margaret Slater

**Affiliations:** 1 Shelter Research and Development, Community Outreach, American Society for the Prevention of Cruelty to Animals (ASPCA^®^), 3201 SW Winding Way, Palm City, FL 34990, USA; 2 Shelter Research and Development, Community Outreach, American Society for the Prevention of Cruelty to Animals (ASPCA^®^), P.O. Box 821075, Kenmore, WA 98028, USA; E-Mail: emily.dolan@aspca.org; 3 Shelter Research and Development, Community Outreach, American Society for the Prevention of Cruelty to Animals (ASPCA^®^), P.O. Box 408, Little Silver, NJ 07739, USA; E-Mail: laurie.garrison@aspca.org; 4 Research and Analytics, Media & Communications, American Society for the Prevention of Cruelty to Animals (ASPCA^®^), 520 Eighth Avenue, 7^th^ Floor, New York, NY 10018, USA; E-Mail: julie.hong@aspca.org; 5 Shelter Research and Development, Community Outreach, American Society for the Prevention of Cruelty to Animals (ASPCA^®^), 50 Stone Ridge Drive, Florence, MA 01062, USA; E-Mail: margaret.slater@aspca.org

**Keywords:** adoption policies, pets as gifts, pet relinquishment, pet attachment

## Abstract

**Simple Summary:**

Policies that state pets should not be adopted as gifts are prevalent at animal welfare organizations, despite the fact that this belief is unfounded. Denying adopters who intend to give the animals as gifts may unnecessarily impede the overarching goal of increasing adoptions of pets from our nations’ shelter system. We found that receiving a dog or cat as a gift was not associated with impact on self-perceived love/attachment, or whether the dog or cat was still in the home. These results suggest there is no increased risk of relinquishment for dogs and cats received as a gift.

**Abstract:**

Policies that state dogs and cats should not be adopted as gifts are prevalent at animal welfare organizations, despite the fact that this belief is unfounded. Denying adopters who intend to give the animals as gifts may unnecessarily impede the overarching goal of increasing the rate of live-releases of dogs and cats from our nations’ shelter system. The results of this brief survey show that receiving a dog or cat as a gift was neither significantly associated with impact on self-perceived love/attachment, nor was it associated with whether or not respondents still had the dog or cat in the home. The results from this survey add to a growing body of literature that suggests there is no increased risk of relinquishment for dogs and cats received as a gift.

## 1. Introduction

Policies that dogs and cats should not be given as gifts are extremely common in the field of animal welfare. Reasons for this come from a wide variety of sources ranging from veterinary organizations like the Oregon Veterinary Medical Association [1] to animal shelters like the Santa Barbara Humane Society [2] to human health resources like WebMD^SM^ [3]. The reasoning behind this policy may, at first glance, seem like common sense. Common arguments include statements such as: pet ownership shouldn’t be an impulse decision; the recipient should consent to pet ownership and be involved in the selection of a pet that fits his or her lifestyle; it’s a long-term commitment; pet ownership is costly; the holidays are a bad time to bring home a new animal. This message may be driven by concerns of inappropriate homing and return to the shelter. Often, organizations that support these arguments cite anecdotal evidence that returns of pets from unhappy gift receivers occurs frequently. However, the available data do not support these concerns. 

Of primary concern is that dogs and cats received as gifts will be relinquished to shelters at a high rate. In fact, the American Humane Association (AHA) estimates that half of all dogs and cats no longer in the home are relinquished to shelters [4]. Studies of dog and cat relinquishment to shelters, however, show that the relinquishment of dogs and cats received as gifts is lower than from other sources. 

In one specific study, New *et al.* [5] identified the source of approximately 2,600 dogs and 2,300 cats relinquished to 12 shelters in four regions of the US. They found that dogs relinquished to shelters had most frequently come from friends, shelters and breeders. Relinquished dogs infrequently came from pet shops, as gifts and from veterinarians. That study found that the odds of dog relinquishment were higher when acquiring an animal from a shelter, friend, as a stray, and from a pet shop (ORs range from 2.1 to 3.1) compared to receiving an animal as a gift (and controlling for other factors such as gender, neuter status, length of ownership and purchase cost), Similarly, cats that were relinquished to shelters had originally come from friends, as strays, and shelters most frequently. Relinquished cats infrequently came from breeders, gifts and veterinarians. The odds of cat relinquishment were higher when acquiring an animal from a shelter, a friend, as a stray, and from a pet shop (ORs range from 1.5 to 3.1) compared to receiving an animal as a gift. These patterns of risk are consistent with previous findings that just looked at source of dogs and cats abandoned because the owner was moving [6]. In addition, Scarlett *et al.* identified 71 reasons given for pet relinquishment [7]. “Unwanted gift” was listed as a reason for only 0.3% of dogs and 0.4% of cats entering the shelters surveyed, compared with “No time for pet” as a reason 10% of dogs were relinquished and “allergies in family” as a reason 18% of cats were relinquished. Finally, Patronek *et al.* [8] examined risk factors for dog relinquishment at one shelter and concluded that dogs that were received as a gift were at significantly decreased risk of being relinquished, compared to dogs who were purchased or adopted. Similarly, cats that were received as a gift were at decreased risk of being relinquished, though this result was not significant [9].

While there is strong existing evidence to show that dogs and cats obtained as gifts are not at a higher risk of relinquishment than dogs and cats obtained in other ways, the myth that dogs and cats should not be given as gifts still persists. The following study was conducted to add to the research about dogs and cats obtained as gifts and aims to examine how receiving a dog or cat as a gift, whether a surprise or not, is associated with the receivers’ self-perceived love or attachment toward the pet and if the gift is associated with the pet still living in the home at the time of the survey. 

## 2. Experimental Section

Data was collected through a larger telephone survey (using both landlines and cell phones) yielding 1,006 adults, 18 years of age and older, living in the continental United States. The survey was conducted from July 18th to 21st, 2013. All interviews were conducted using ORC International’s (ORC) computer assisted telephone interviewing (CATI) system. Phone numbers were identified using list-assisted Random Digit Dialing. The research questions addressed in this report are based on a subsample of 222 of the 1,006 total respondents. These 222 respondents are those who answered “yes” to the question “Have you received a pet dog or cat as a gift in the past 10 years or so?”.

### 2.1. Items from Survey

In addition to pertinent demographic information, we report the results from three questions about receiving their dogs and cats as a gift:

- **Involvement in the selection of the pet:** Were you involved, in any manner, in the selection process of the pet that you received as a gift? Response options were: Yes, you were involved; No, you were not involved but you wish you were involved; Or, no, the pet was a surprise and that was okay.
- **Attachment to pet:** Did receiving a pet as a gift increase, decrease or have no impact on your love or attachment to the pet? Response options were: increased my love or attachment to the pet; decreased my love or attachment to the pet; did not make any difference.
- **Duration of ownership:** Is that pet still with you? Response options were: Yes; No, he or she passed away; Or, no, the pet has another home.

### 2.2. Data Analysis

All statistical procedures were conducted using Stata/IC 13.0 (StataCorp., College Station, TX, USA). We calculated frequencies for tables. Because the expected value of some of the cells was less than five, we used Fisher’s exact test to examine associations between receiving a pet as a surprise gift or not and the extent to which the gift impacted love/attachment and whether the pet was still in the home. 

## 3. Results and Discussion

### 3.1. Demographics

Two hundred and twenty two total respondents said they received a dog or cat as a gift in the past 10 years or so. Amongst those respondents, 128 (57.7%) were female, 195 (87.8%) reported being head of household, 102 (46.0%) reported some employment, 117 (52.7%) reported being married or living with a partner, 147 (66.2%) owned their own home, 62 (27.9%) had children under the age of 18 living in the home, 200 (90.1%) completed high school or higher, 24 (10.8%) reported being of Hispanic origin, and 168 (75.7%) reported being white. The majority of respondents, 119 (53.6%), reported a total household income less than $50,000 per year, and 125 (56.3%) reported being younger than 55 years old. Respondents were spread throughout various regions of the country: New England (13.1%), Mid West (21.6%), South (39.6%) and West (25.7%). 

The frequencies in Table 1 and Table 2 reveal that most respondents reported that they were either involved in the gift selection or they were surprised, but being surprised was ok with them. The vast majority of respondents in Table 1 reported that receiving the animal as a gift either increased their self-perceived love/attachment for the dog or cat or that it did not make a difference. Only seven respondents reported that receiving the animal as a gift decreased their self-perceived love/attachment. Table 2 shows that the majority of respondents report that the dog or cat was still in the home at the time of the survey, regardless of how the pet was acquired. 

**Table 1:** Frequencies of the impact receiving a gift has on self-perceived love or attachment. * Response choices: Refused, Don’t Know and Other are excluded.

		Impact on self-perceived love or attachment			
		Increased	Decreased	No Difference	Total *
		n (row %)	n (row %)	n (row %)	
**Levels of Involvement in pet selection**	Surprised, but it was ok	72(59.5)	5(4.1)	44(36.4)	121
	Surprised, wish involved	11(64.7)	0(0.0)	6(35.3)	17
	Involved/not surprised	43(53.8)	1(1.3)	36(45.0)	80
	Total *	126	6	86	218

**Table 2:** Frequencies of the impact receiving a gift has on retention in the home. * Response choices: Refused, Don’t Know and Other are excluded.

		Pet still in the home			
		Yes	No, died	No, rehomed	Total *
		n (row %)	n (row %)	n (row %)	
**Levels of Involvement in pet selection**	Surprised, but it was ok	92(76.7)	13(10.8)	15(12.5)	120
	Surprised, wish involved	13(76.5)	3(17.7)	1(5.9)	17
	Involved/not surprised	64(82.1)	9(11.5)	5(6.4)	78
	Total *	169	25	21	215

Fisher’s exact test revealed that receiving the dog or cat as a gift was not significantly associated with impact on self-perceived love/attachment (*p* = 0.59) nor was it associated with whether or not respondents still had the dog or cat in the home (*p* = 0.59). Because these data came from a survey, we considered the possibility that the sampling method of the survey may have introduced error into our findings. We therefore calculated the tables using weighted values adjusting for age, sex, region, race/Hispanic origin and education. The weighted results did not demonstrate a different pattern and so are not shown here, in favor of a simpler presentation. Further, because Fisher’s test does not account for weighting, we combined the frequencies for self-perceived love/attachment so that the expected value in each cell was above five (We were unable to combine the home retention table such that the expected value of each cell was above five). We ran a weighed logistic regression of receiving a gift on self-perceived love/attachment and did not find a significant relationship between the two variables (results not shown). 

### 3.2. Discussion

This survey showed no significant relationship between receiving a dog or cat as a gift, whether a surprise or not, and the receivers’ self-perceived love or attachment toward the pet. Nor was there a significant relationship between receiving a dog or cat as a gift and whether the pet was still living in the home at the time of the survey. These data supports previous research by New *et al.* and others [5,6,7,8,9] that suggests receiving a dog or cat as a gift does not negatively impact that pet, either by altering the human-pet bond, or by shortening the time the pet is kept in the home. While much of the historic data in this area focused on relinquished animals, this data set focused on those who may still have the dog or cat in their home.

In addition to evidence that receiving an animal as a gift is associated with a lower risk of relinquishment [8,9], a growing body of evidence seems to go against a related idea that research and planning are an important part of successful pet ownership. Research from the AHA [4] found that dogs and cats obtained spur of the moment or with little thought compared to dogs and cats obtained after lengthy research were not more likely to be relinquished to animal shelters. These data are similar to the Patronek findings that cats unexpectedly acquired were at decreased risk of relinquishment [9] and there was no association between planning and relinquishment for dogs [8]. 

Our sample is generally comparable to the average type of dog or cat owner. A 2012 American Veterinary Medical Association (AVMA) report indicates that dog or cat owners are largely female, work full time, are married, own their own home, have more than one or two family members living in the house, do not have advanced degrees, are white and about half have income lower than $55K [10]. This is comparable to the AHA report which found that most household respondents had two adults, 25% of which had children, 75% of which were white [4].

These findings may help animal welfare organizations open options for those interested in obtaining dogs and cats for their family and friends. It is important to note that animals obtained from a shelter are more at risk than those obtained as gifts [5]. Allowing adoptions of dogs and cats to those obtaining the pet as a gift may decrease the risk of return or relinquishment for that dog or cat. Furthermore, it would allow for more animals from shelters to find homes. 

In addition to potentially improving the adoption rate of dogs and cats from shelters, there are benefits conferred to individuals and families by gifting animals. In one particular opinion piece published in Childhood Education, Shirley O’Brien goes so far as to recommend the gift of a dog or cat in order to teach children about security, self-esteem and responsibility, unconditional and open love; necessary constructs for children’s development [11]. 

### 3.3. Limitations

We did not identify how long the dog or cat lived in the home to which it was gifted. We can assume from the selection criteria question that it is somewhere between 1 day and 10 years between the time of the gifting and the completion of the survey. The survey represents a small, cross-sectional sample and was not conducted to solely answer this question, thus, the generalizability of these findings may be limited. Further, because this was a secondary data analysis, our measure of attachment did not come from a validated scale, but represents a subjective, self-perceived change in “love or attachment”.

## 4. Conclusions

The lack of associations between receiving a dog or cat as a gift and its impact on self-perceived love/attachment and whether or not respondents still had the dog or cat in the home is evidence supporting the notion that pets given as gifts are not at higher risk for abandonment. Further, denying potential adopters the right to obtain dogs and cats as gifts may unnecessarily impede the overarching goal of increasing the rate of live-releases of dogs and cats from our nations’ shelter system. 
